# Metabolic Effects of Known and Novel HDAC and SIRT Inhibitors in Glioblastomas Independently or Combined with Temozolomide

**DOI:** 10.3390/metabo4030807

**Published:** 2014-09-12

**Authors:** Miroslava Cuperlovic-Culf, Mohamed Touaibia, Patrick-Denis St-Coeur, Julie Poitras, Pier Jr Morin, Adrian S. Culf

**Affiliations:** 1 National Research Council of Canada, 100 Rue des Aboiteaux St., Moncton, NB E1A 7R1, Canada; 2 Department of Chemistry and Biochemistry, Université de Moncton, Moncton, NB E1A 3E9, Canada; E-Mails: mohamed.touaibia@umoncton.ca (M.T.); eps7653@umoncton.ca (P.-D.S.-C.); ejp6815@umoncton.ca (J.P.); pier.morin@umoncton.ca (P.J.M.); 3 Atlantic Cancer Research Institute, Moncton, NB E1C 8X3, Canada; E-Mail: AdrianC@canceratl.ca

**Keywords:** metaboloepigenomics, NMR metabolomics, glioblastoma, enzyme inhibitors, combined treatment

## Abstract

Inhibition of protein deacetylation enzymes, alone or in combination with standard chemotherapies, is an exciting addition to cancer therapy. We have investigated the effect of deacetylase inhibition on the metabolism of glioblastoma cells. ^1^H NMR metabolomics analysis was used to determine the major metabolic changes following treatment of two distinct glioblastoma cell lines, U373 and LN229, with five different histone deacetylase (HDAC) inhibitors, as well as one inhibitor of NAD+-dependent protein deacetylases (SIRT). The addition of the standard glioblastoma chemotherapy agent, temozolomide, to the HDAC and SIRT treatments led to a reduction in cell survival, suggesting a possibility for combined treatment. This study shows that distinct glioblastoma cell lines, with different metabolic profiles and gene expression, experience dissimilar changes following treatment with protein deacetylase inhibitors. The observed effects of inhibitors on mitochondrial metabolism, glycolysis and fatty acid synthesis suggest possible roles of protein deacetylases in metabolism regulation. Metabolic markers of the effectiveness of anti-protein deacetylase treatments have been explored. In addition to known deacetylation inhibitors, three novel inhibitors have been introduced and tested. Finally, ^1^H NMR analysis of cellular metabolism is shown to be a fast, inexpensive method for testing drug effects.

## 1. Introduction

Glioblastoma multiforme (GBM) is the major, aggressive form of brain tumour in adults. In spite of some advances in therapy, sufferers of this tumour type still have a very poor prognosis, with fewer than 5% of patients surviving the five-year mark [1]. Patients diagnosed with a GBM generally undergo surgical resection followed by radiotherapy with concurrent and adjuvant treatment using the chemotherapeutic agent, temozolomide (TMZ) [2]. Improved, as well as combined therapies for GBM are thus sorely needed. Several novel, exploratory anticancer drugs attempt to alter tumour cell properties by modulating gene expression or activation of proteins, making tumours more susceptible to chemotherapy or radiation therapy. An interesting avenue in this approach is based on the observation that many tumours, including GBMs, show hypoacetylation of histones H3 and H4 and overexpression of histone deacetylases [3]. Acetylation of lysine residues of proteins is a reversible modification regulated by the antagonistic activity of two groups of enzymes: histone acetyl transferases (HAT) and histone deacetylases (HDACs and SIRTs). Changes in histone deacetylase expression lead to altered gene expression, as well as differences in protein activation in tumour cells. Therefore, histone deacetylase inhibitors are being explored as promising agents and enhancers of anti-cancer therapy. Amongst many proposed HDAC inhibitors, vorinostat also known as suberanilohydroxamic acid (SAHA) and depsipeptide FK228 (romidepsin) have been approved for treatment of cutaneous T-cell lymphoma, and their use in other tumours is currently being evaluated in clinical trials. Treatment with HDAC inhibitors appears to induce apoptosis via the activation of caspases [4,5,6] or through autophagy [7,8,9]. In laboratory tests, SAHA has been shown to activate autophagy in GBMs by inhibiting mTOR and upregulating LC3 expression [9]. Clinical analysis of the effect of SAHA on GBM patients showed only modest, if any, effect on overall patient survival [10,11]. However, the addition of HDAC inhibitors to chemotherapy or radiation therapy has been proven beneficial in early studies [12,13,14,15]. Although all GBM patients generally receive the same treatment, the molecular characteristics of GBMs are highly diverse, with significant differences in the expression and activity of a number of tumour markers, including differences in HDAC expression levels and differences in expression, concentration and activation of their regulators, as well as targets [16,17]. With histone deacetylases and metabolism mutually regulated, *i.e.,* in regulatory interactions, it can be expected that histone deacetylase activity will be reflected in metabolic changes and that metabolic differences in tumours will affect histone deacetylase activity. It is now well established that tumour cells show a remarkably different metabolism than the tissues that they derive from. A number of oncogenes converge to create this altered metabolism in order to support the growth and survival of cancer cells under suboptimal conditions [18,19]. Amongst factors creating metabolic change are the epigenetic factors. At the same time, metabolic changes in cells have an effect on epigenetic changes, including protein acetylation [20].

A handful of studies exploring the effect of histone deacetylase inhibitors on cellular metabolism have shown profound effects. A recent report has shown that SAHA treatment of breast cancer cell lines leads to elevated levels of phosphocholine [21]. This follows earlier studies similarly showing that HDAC inhibitors treatment leads to increased phosphocholine levels in cells and tumours *in vivo* [22,23]. In breast cancer cell lines, this change is assigned to the increased expression of choline transporter SLC44A1 [24], as well as increased expression and activity of choline kinase following application of SAHA [21]. In neuronal cells, the HDAC inhibitor, trichostatin A, decreases cholesterol levels by inducing the expression of genes involved in cholesterol efflux and catabolism and reducing the expression of genes involved in synthesis [25]. A study by Wardell and co-workers [26] has indicated that non-selective inhibition of HDACs (SAHA and valproate) in multiple myeloma cells quantitatively inhibits glucose transporter 1 (GLUT1)-mediated glucose transport through downregulation of GLUT1 and inhibition of hexokinase 1, a major glycolytic enzyme. Furthermore, these authors have shown that HDAC inhibition leads to increased use of amino acid catabolism as an energy and carbon source in cancer cells. In less proliferative lung cancer cells (H460), pan-HDAC inhibition with sodium butyrate and trichostatin A increases mitochondrial function and oxidative metabolism [27]. In addition, specific loss of HDAC6 results in reduced mitochondrial metabolic activity through reduction in the net activity of mitochondrial enzymes, including respiratory complex II and citrate synthase [28]. One of the major functions of SIRTs is the regulation of cellular metabolism in response to nutrient availability [29] through, at least in part, regulation of acetyl-CoA synthase [30]. Furthermore, SIRT6 is a specific deactivator of HIF1a and MYC expression that can lead to the suppression of aerobic glycolysis and anabolic glutamine metabolism [31,32].

Distinct GBM cell lines exhibit differences in metabolic profiles due to variations in isocitrate dehydrogenase (IDH1) expression [33] and in other factors, such as epidermal growth factor receptor (EGFR) [34]. In this work, we have set out to determine the metabolic effects of a variety of HDAC and SIRT inhibitors on the metabolism of two distinct GBM cell cultures. Cell cultures selected for these novel experiments represent two groups of GBMs with previously demonstrated distinct metabolic profiles [34]. HDAC inhibitors are generally viewed as an addition to other chemotherapy treatments, including DNA-damaging chemotherapeutic agents, radiotherapy, hormonal therapies, DNA methyltransferase inhibitors, as well as other small molecule inhibitors [35]. Therefore, we have also explored the effects on the cell survival and metabolism of combined treatment with HDAC or SIRT inhibitors and TMZ.

## 2. Results and Discussion

**Figure 1:**
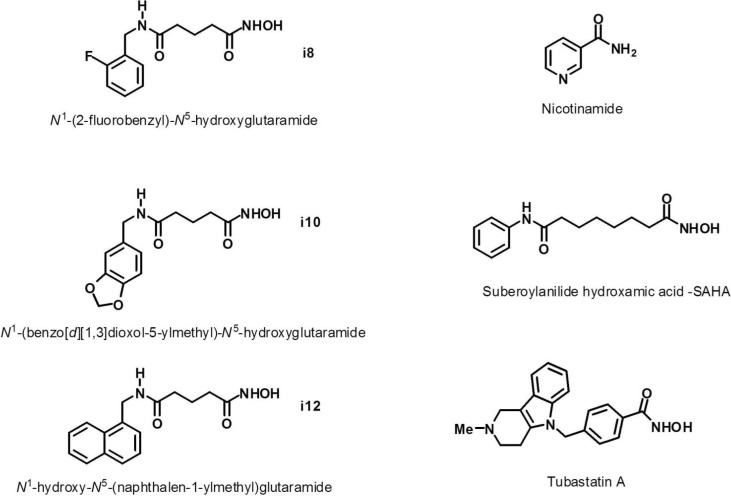
Chemical structures of compounds used for the HDAC and SIRT inhibition explored in this work.

In this work, we have set out to determine the metabolic effects of five inhibitors of HDACs and one of the SIRTs on the GBM cell lines. The structures of these compounds are shown in Figure 1. Compounds listed as i8, i10 and i12 are moderately selective class IIb HDAC6 inhibitors recently designed and synthesized by members of our group [36]. Nicotinamide (Nico) is a known inhibitor of sirtuins, class III NAD^+^-dependent histone deacetylases [37,38]. SAHA (Vorinostat) is a pan-HDAC inhibitor currently approved for cancer therapy [39,40,41]. Although SAHA is generally accepted as a pan-HDAC inhibitor, it is particularly potent against HDACs 1, 2, 3 and 6 [9,40,41] with possibly some selectivity towards class I HDACs, particularly HDAC1 and HDAC8 [42]. Tubastatin A (TUBA) is a highly selective class IIb inhibitor with strong activity against HDAC6 [43]. An ability of these compounds to cross the blood-brain-barrier is predicted using Lipinski “rule-of-five” criteria as reviewed in Pajouhesh and Lenz [44]. The five properties included in the “rule-of-five” requirements are listed in Table 1. According to this prediction method, all seven compounds have drug-like characteristics. Negative cLogP values of TMZ and nicotinamide suggest hydrophilic behaviour. It is interesting to point out that although TMZ is the standard GBM therapy drug, it has been previously shown to have a very low cerebrospinal fluid to plasma ratio of 22.7% [45]. The calculated LogP value for TMZ (shown in Table 1) is in alignment with this experimental result. The newly introduced compounds, i8, i10 and i12, are more lipophilic and fit within the range of known central nervous system drugs according to all five properties listed in Table 1.

**Figure 2:**
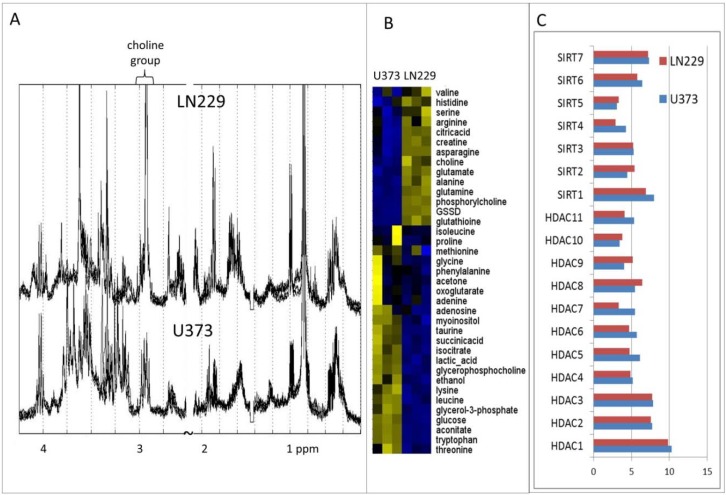
Overall metabolic makeup of U373 and LN229 glioblastoma multiforme (GBM) cell lines used in this study. (**A**) Average 1H 1D NMR spectra of cell lines LN229 and U373 exposed to DMSO. Shown is the average value of three biological replicates. (**B**) Relative metabolite concentrations for intracellular metabolites in two cell lines measured in three biological replicates. Metabolite concentrations have been scaled (across samples) and normalized (across metabolites). (**C**) Relative gene expressions for all HDAC and SIRT genes in LN229 and U373 cells (from microarray data provided in [49]). Shown are the values obtained following sample normalization and averaging of measurements for each gene.

The effect of these inhibitors was tested on two distinct GBM cell lines, LN229 and U373, selected for their different response to TMZ. Furthermore, these cell lines have been shown to possess different metabolic profiles [34], and this observation is confirmed here with new metabolic measurements (Figure 2A,B). An evident distinction between these two cell lines is in the expression level of epidermal growth factor receptor (EGFR) [34]. EGFR is a transmembrane glycoprotein that binds to epidermal growth factor. Amongst its many functions, EGFR has been linked to the mechanism that reduces cell membrane permeability to choline [46,47]. Metabolomics analysis supports this role of EGFR, where U373 cells have a significantly lower concentration of choline and phosphorylcholine in the cytoplasm (Figure 2). Another well-established distinction between LN229 and U373 cells is in the expression levels of enzymes involved in the arachidonic acid pathway, once again leading to significant metabolic alterations [48]. Genetic and transcriptomics differences between these two cell lines have been explored by Wiedemeyer *et al*. [49], showing differential expression for the number of other genes, as well as mutation in phosphatase and tensin homolog (PTEN) in U373 cells. PTEN is an enzyme involved in the removal of phosphate groups from proteins and lipids, thereby directly affecting metabolism. These and possibly other differences are clearly emphasized by highly significantly dissimilar concentration levels for the majority of metabolites visible in the experiments presented here (Figure 2). Metabolic differences between these two and a number of other GBM cell lines were a focus of [34]. LN229 and U373 cell cultures show distinct choline metabolism, mitochondrial metabolism, as well as amino acid metabolism, particularly in branched chain amino acids. However, according to publically available microarray data, HDACs’ and SIRTs’ expression levels in the two cell lines are not significantly different. Figure 2C shows relative expression levels for all HDAC and SIRT genes in LN229 and U373 cells (obtained from gene expression data for GBM cell lines and tissues provided in [49]). Although, gene expression can be a factor in the regulation of the activity of HDACs and SIRTs, their functions are significantly affected by interactions with many partner proteins [50] that can have different expression and/or activation levels.

**Table 1:** Properties of the tested compounds in relation to the “rule of five” characterization of drugs [44]. Hydrophobicity is represented by the logarithm of the partition coefficient for n-octanol/water LogP (cLogP) calculated using ChemDraw Ultra 10.0 (cLogP). The allowed limits for a molecule to be considered as a drug are shown in brackets and indicated with *. The ranges observed for all known central nervous system drugs are indicated with # in the table. TMZ, temozolomide; TUBA, tubastatin A, SAHA - vorinostat also known as suberanilohydroxamic acid.

Compound	cLogP (≤5)^*^ (0.16–6.59)^#^	MW (≤500)^*^ (151–655)^#^	H-Bond Donors (∑OHs + ∑NHs) (≤5)^*^ (0–3)^#^	H-Bond Acceptors (∑Ns + ∑Os + ƩFs) (≤10)^*^ (1–10)^#^	No. Rotatable Bonds (≤10)^*^ (0–5)^#^
TMZ	−0.81	194.15	2	8	3
i8	0.28	254.26	3	6	8
i10	0.10	280.28	3	7	8
i12	1.31	286.33	3	5	8
Nicotinamide	−0.21	122.12	2	3	2
SAHA	0.99	264.32	3	5	10
TUBA	2.38	335.4	2	5	6

**Figure 3:**
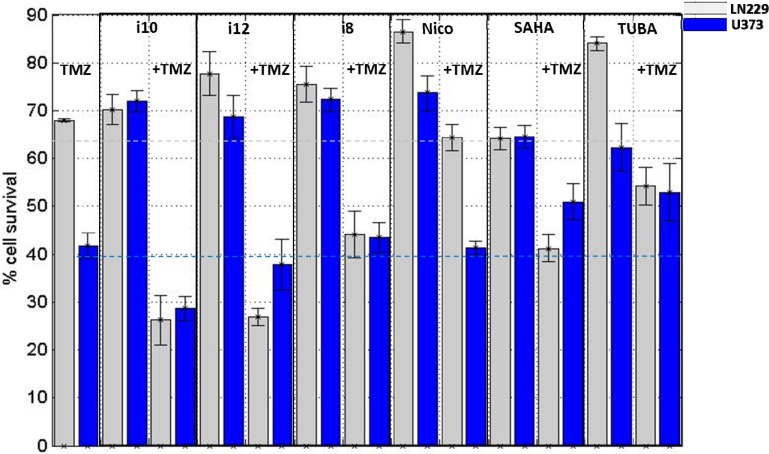
Cell survival measurements following exposure of cells to temozolomide (TMZ) or compounds i10, i12, i8, nicotinamide (Nico), SAHA and tubastatin A (TUBA). Shown are the cell survival measurements for individual treatment, as well as combined cell treatment with TMZ and studied compounds. Error bars show the standard error of the mean for three replicated measurements. The same amounts of compounds were used for treatment in two cell lines (amounts listed in Table 2). Cell survival measurements were performed following 72 h treatments.

These two cell types were treated with inhibitors, either alone or in combination with TMZ. Compound concentrations used for crystal violet assays (cytotoxicity) are provided in Table 2. Cell survival levels following these treatments are shown in Figure 3.

**Table 2:** Compound concentrations used for crystal violet assays (cytotoxicity) and for treatment for metabolomics analysis. Cell survival was measured following 72 h incubation.

Compound	Concentration (µM)
TMZ	250
i8	20
i10	20
i12	20
Nicotinamide	5 mM
SAHA	0.5
TUBA	0.5

The compound concentrations used for cell survival measurements (Table 2) were also used for treatment of U373 and LN229 cell lines for metabolomics analysis. These concentrations corresponded to approximately 50% of the calculated IC_50_ concentrations for individual compound exposure of U373 cell lines. Low concentrations were used in order to identify metabolic changes caused by initial drug action rather than those induced by cell death or secondary effects. For consistency, the same compound concentrations were utilized for the treatment of both cell lines. All cytotoxicity measurements were performed in triplicates, and the standard errors of mean are shown as error bars in Figure 3. Cell survival levels were in all cases for both cell lines lowered by the addition of TMZ. U373 cells are susceptible to TMZ treatment, having less than 50% of cells surviving treatment. LN229 cells are more resilient to TMZ treatment with almost 70% of cells surviving equivalent TMZ treatment. Transcript levels of MGMT, a key molecular target underlying TMZ resistance, are comparable in these cell models according to RT-PCR measurements. Hence, differences in TMZ sensitivity in U373 and LN229 cells are likely attributable to differential expression levels of other players responsible for TMZ resistance, such as MSH6 and MLH1 [51,52,53]. In the future, a more detailed molecular picture associated with TMZ resistance in LN229 and U373 cells needs to be generated. Combinatory treatment of LN229 cells with histone deacetylase inhibitors and TMZ significantly reduced cell survival. In TMZ-susceptible U373 cell culture, histone deacetylase inhibitors did not induce larger cell death than TMZ treatment alone. Strikingly, even the addition of TMZ to HDACi and SIRTi treatments for U373 cells did not increase cell death above the levels observed when using TMZ alone (except for i10). In fact, for U373 cells combining TUBA and SAHA with TMZ treatment increased cell survival relative to the observed cell survival following TMZ exposure alone. This observation is in agreement with Kitange *et al*. [54], who have recently indicated that treatment of GBM flank xenografts with TMZ and SAHA favors the evolution of TMZ resistance by MGMT overexpression as compared with treatment with TMZ alone. Determination of specific mechanisms for this epigenetically-directed therapy resistance to TMZ will require further investigation.

**Figure 4:**
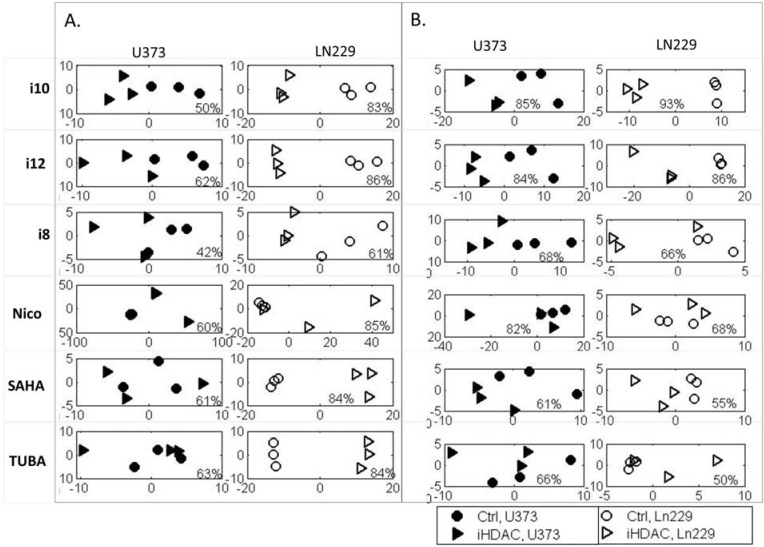
PCA of ^1^H 1D NMR spectral data for two cell lines treated with the studied compounds and controls (DMSO). Each treatment has been done in triplicate. PCA has been performed and shown separately for two cell lines and for each compound treatment with the control. (**A**) The analysis of the measurements of intracellular metabolites is shown, and (**B**) the corresponding extracellular samples’ analysis is presented. Each PCA plot includes information about the percentage of variance described by Principal Component 1 (PC1).

Histone deacetylases and the inhibition of histone deacetylases have a profound effect on cancer cell metabolism through the action of HDACs on histones and gene expression, as well as through direct activation of a number of proteins (for a recent review, see [55]). In order to determine the relationship between histone deacetylases and cancer cell metabolism, we performed metabolomics analysis of GBM cells treated with histone deacetylase inhibitors (Figure 1). Treatments were applied to two cell lines in triplicate, and the metabolic extracts for hydrophilic intracellular and extracellular metabolites were prepared for ^1^H NMR analysis as previously described [34,47,48,56] The resulting 156 NMR spectra are available from the authors. Principal component analysis (PCA) of non-binned, aligned spectra was used to explore overall separation of treated and control samples in LN229 and U373 cells for both intracellular (Figure 4A) and extracellular (Figure 4B) metabolites. The figures include analysis of metabolic extracts of cells treated with HDAC and SIRT inhibitors only. Metabolic changes are even more profound in cells treated with inhibitors together with TMZ (available upon request). Each plot includes information about the percentage of variance that is described by Principal Component 1 (PC1). For the majority of tested compounds, PC1 includes over 50% of spectral variance, suggesting that the separation of samples observed across PC1 is highly significant.

According to PCA of intra- and extra-cellular metabolites, treatments with i10, i12, i8, SAHA and TUBA cause major metabolic shifts in cellular metabolism, as well as in metabolite transport in LN229 cells. The overall metabolic profile of U373 cells appears to be affected the most by nicotinamide (Nico), as suggested by the largest separation of treated and control samples by PCA of intracellular metabolites. In U373 cells, metabolic changes from other treatments are only visible in the extracellular medium, suggesting that cells maintain their internal processes by an increase in metabolites transport to and from the extracellular medium. This observation would suggest a more significant role for HDACs in the metabolism of LN229 cells and more involvement of SIRT in the regulation of U373 cells metabolism.

**Figure 5:**
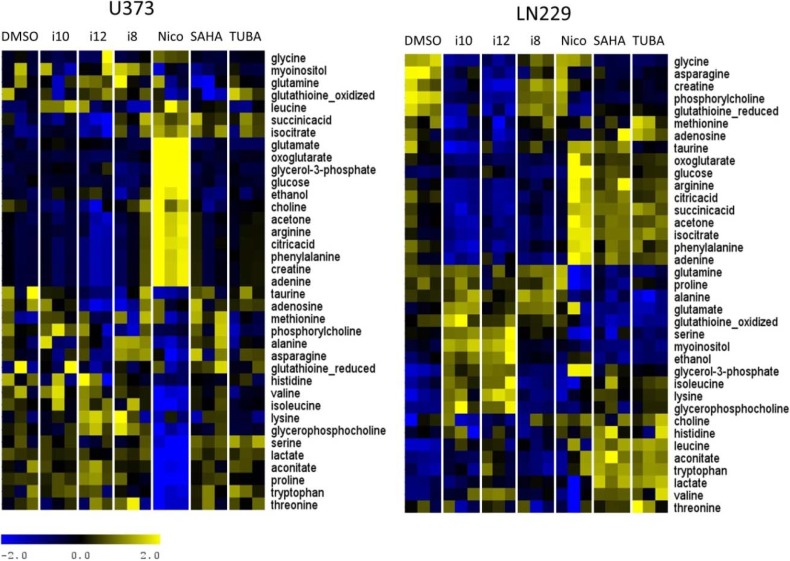
Relative metabolite concentration for intracellular samples for two cell lines treated with the studied compounds. Concentrations are scaled across samples and then normalized across metabolites. Heat maps shown for the two cell lines have been clustered across metabolites for easier visualization.

**Figure 6:**
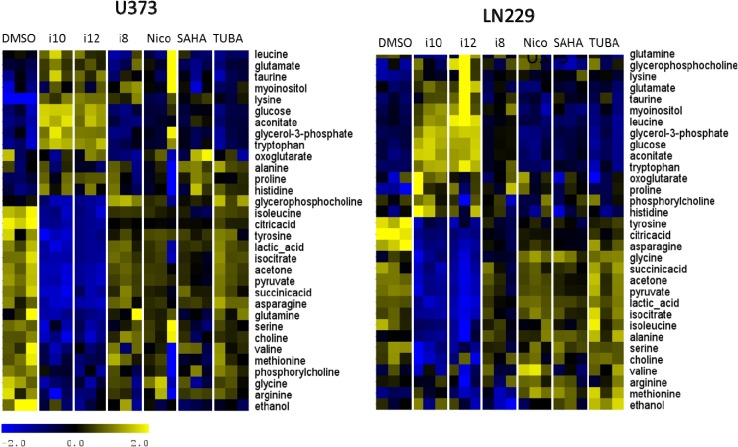
Relative metabolite concentrations for extracellular samples for two cell lines treated with all individual compounds. Concentrations are scaled across samples and normalized across metabolites. Heat maps shown for the two cell lines have been clustered across metabolites for easier visualization.

Metabolite quantification from NMR spectra was performed using a method developed in our group [34]. From the hydrophilic, intracellular samples, it was possible to obtain the relative concentrations for 37 metabolites, and the extracellular medium provided information for 32 metabolites. PCA of quantitative metabolic data is in close agreement with the PCA of corresponding spectral data. Relative metabolite concentrations in U373 and LN229 cell extracts are shown in Figure 5 and Figure 6. Figure 5 shows concentrations of metabolites in the intracellular extracts and Figure 6 shows concentrations of metabolites in the extracellular medium.

Relative metabolite concentration differences between distinct treatments shown in Figure 5 and Figure 6 show large metabolic changes with nicotinamide treatment in U373 cells, the major influence of i10 and i12 on the metabolism of both cell lines, as well as more a significant influence of SAHA and TUBA on intracellular metabolites in LN229 cells.

**Figure 7:**
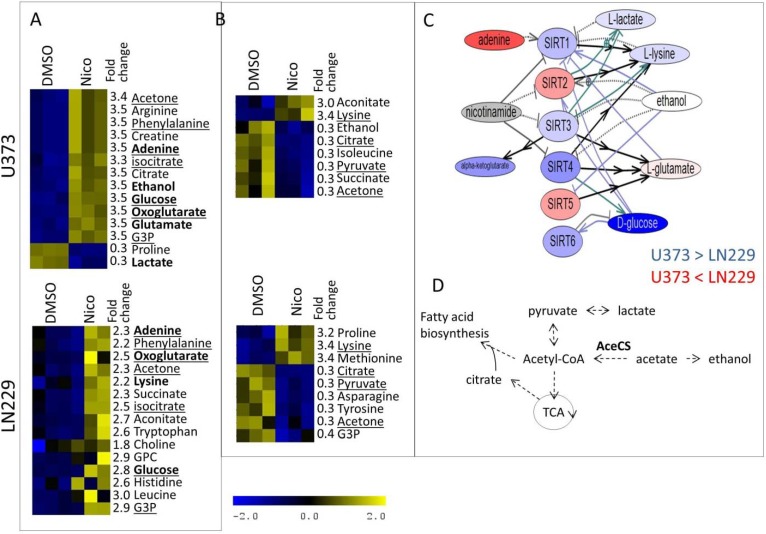
Major metabolic differences induced by nicotinamide (Nico) treatment in intracellular (**A**) and extracellular (**B**) samples. Metabolites that are determined as the most significantly changed by treatment in both U373 and LN229 cells are underlined. Metabolites are shown in (C) and (D) in bold. (**C**) Pathway Studio determined literature-based relationships between different members of sirtuin family (SIRT) and select metabolites. Gene expression values were obtained from publicly available microarray measurements [49]. Shown are metabolite concentrations in control samples (DMSO treatment). For both genes and metabolites, blue corresponds to a higher concentration in U373 cells relative to LN229 cells and red represents higher concentration in LN229 cells, with darker colors indicating a larger relative difference. Ethanol (white) is equally concentrated in two cell lines. The concentration of nicotinamide is not available from NMR metabolic data (gray), although in the treatments, the same concentration of nicotinamide is used. (**D**) Schematic representation of some metabolic processes regulated by SIRT according to literature information that includes some of the significantly changed metabolites according to the data shown here.

**Figure 8:**
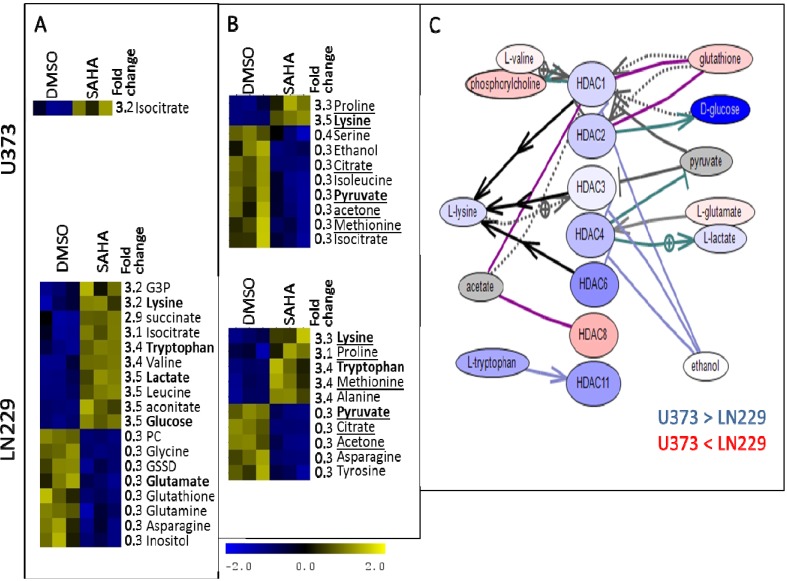
Major metabolic differences induced by SAHA treatment in intracellular (**A**) and extracellular (**B**) samples. Metabolites that are most significantly changed by treatment in U373 and LN229 cells are underlined. Metabolites that are shown in Figure 8C are shown in bold. (**C**) Pathway Studio determined literature-based relationships between different HDACs and select metabolites. Gene expression values were obtained from publicly available microarray measurements [49] Shown are metabolite concentrations in control samples (DMSO treatment). For both genes and metabolites, blue corresponds to a higher concentration in U373 cells relative to LN229 cells and red represents higher concentration in LN229 cells, with darker colors indicating a larger relative difference. Ethanol (white) is equally concentrated in two cell lines. The concentrations of acetate and pyruvate are not available (gray).

**Figure 9:**
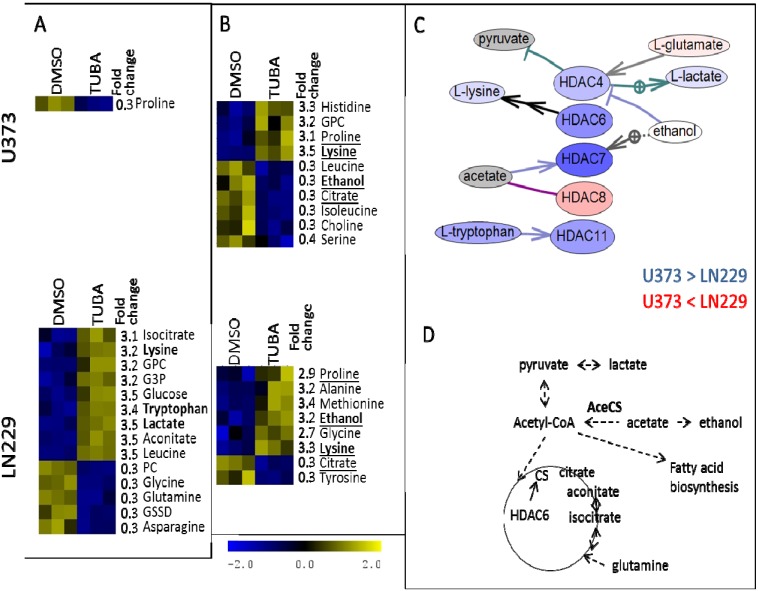
Major metabolic differences induced by tubastatin A (TUBA) treatment in intracellular (**A**) and extracellular (**B**) samples. Metabolites that are most significantly changed by treatment in U373 and LN229 cells are underlined. Metabolites that are shown in Figure 9C and Figure 9D are in bold. (**C**) Pathway Studio determined literature-based relationships between different HDACs and select metabolites. Gene expression values were obtained from publicly available microarray measurements [49]. Shown are metabolite concentrations in control samples (DMSO treatment). For both genes and metabolites, blue corresponds to a higher concentration in U373 cells relative to LN229 cells and red represents higher concentration in LN229 cells, with darker colors indicating larger relative difference. Ethanol (white) is equally concentrated in two cell lines. The concentrations of acetate and pyruvate are not available (gray). (**D**) Schematic representation of some metabolic processes that can be affected by HDAC6 inhibition according to literature information and confirmed in the metabolomics measurements.

**Figure 10:**
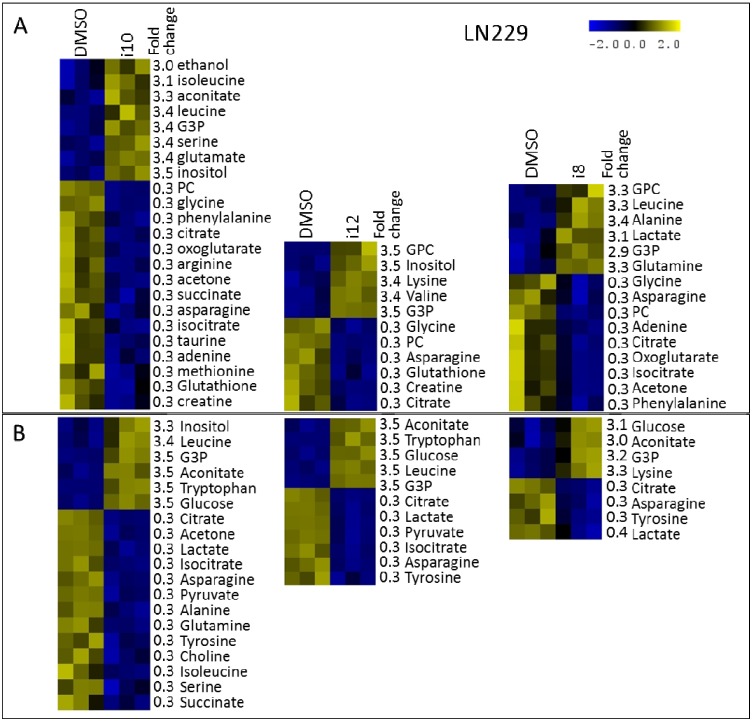
Major metabolic differences induced by i10, i12 and i8 treatments in intracellular (**A**) and extracellular (**B**) metabolites.

Metabolites with major, statistically significant changes between control and treated samples are determined using significance analysis methods (SAM) provided by Tusher, *et al.* [57] and previously introduced as a method for metabolomics analysis by our group [34,47,48,56]. These specific, substantial metabolic differences for each treatment and each cell line are shown in Figure 7, Figure 8, Figure 9 and Figure 10.

Figure 7 shows significant differentially concentrated metabolites in U373 and LN229 cells treated with nicotinamide, as determined from the analysis of intracellular (A) and extracellular samples (B). Also shown is the relationships between members of sirtuin family and the subset of metabolites obtained from high throughput literature search (“bibliomics”) using Pathway Studio (C). In Figure 7C, gene and metabolite colours indicate expression levels in U373 relative to LN229 cells, where blue represents over-concentration or overexpression in U373 cells relative to LN229. Gene expressions were determined from previously published microarray data [49]. Finally, a schematic representation of a subset of metabolic pathways that are regulated by SIRTs is shown in Figure 7D.

Nicotinamide inhibits all sirtuins to some extent [58], likely resulting in changes in the activity of multiple proteins. However, according to our IC_50_ tests (Figure 3 and Table 1), cell death is only achieved with high concentrations of nicotinamide. U373 cells are more affected by nicotinamide than LN229 cells based on the cytotoxicity assay results. The effect on U373 cells is even more evident in the metabolic profiles shown in Figure 4 and Figure 5. As one of SIRTs’ major roles is the regulation of metabolism [29], it is not surprising that cells show a shift in their metabolic preferences following SIRT inhibition, even without significant cell death. However, it is important to notice that U373 and LN229 cell lines experience some distinct metabolic changes. In U373 cells, there appears to be an increase in oxidative phosphorylation with increased levels of Krebs cycle intermediates, as well as reduced lactate production and pyruvate export out of the cell. No change in choline levels is observed in U373 cells. SIRT1, SIRT3 and SIRT4 appear to be slightly more expressed in the U373 cell line relative to LN229 according to previous microarray experiments (Figure 2C and Figure 7C using data from Wiedemeyer *et al.* [49]). Inhibition of mitochondrial SIRT3 would likely impact TCA cycle activation the most, and changes in TCA cycle metabolites are apparent. At the same time, cytoplasmic SIRT1 can influence glycolysis, as well as lipid synthesis, possibly represented by changes in the concentration levels of citrate, glycerol-3-phosphate and glutamate. In LN229 cells, the major increases observed are in choline concentrations (with both choline and glycerophosphocholine concentrations increasing) and glycerol-3-phosphate, possibly due to changes in lipid metabolism. Increases in levels of TCA cycle metabolites are also observed in LN229 cells, even though this is not the case for lactate. Both cytoplasmic and mitochondrial acetyl-CoA synthase (AceCS, Figure 7D) enzymes are inactive when acetylated. Therefore, inhibition of SIRTs, as their deacetylases, would deactivate AceCSs, leading to reduced production of acetyl-CoA from acetate [30]. In this scenario of reduced AceCS activity, pyruvate from glycolysis would preferentially enter the TCA cycle, possibly leading to the observed increase in several TCA cycle intermediates (oxoglutarate, isocitrate, aconitate and succinate). Furthermore, acetate would possibly be metabolized into ethanol rather than acetyl-CoA, possibly leading to the observed increase in ethanol concentration in U373 cells. Altered levels of acetyl-CoA would likely affect fatty acid biosynthesis, possibly resulting in the observed increase in choline and glycerol-3-phosphate levels in LN229 cells. Both choline and glycerol-3-phosphate are involved in the choline phospholipid pathway that is known to be significant in tumour cells [59]. Choline, choline derivatives and glycerol-3-phosphate are also factors in *de novo* fatty acid synthesis, one of the pathways regulated, at least in part, by SIRT [60] and acetyl-CoA levels. Finally, in mammalian cells, nicotinamide can be produced from tryptophan through the activity of SIRT1-7 amongst other factors [58]. Thus, an increase in tryptophan levels in LN229 cells might be due to reduced tryptophan utilization for nicotinamide production.

Figure 8 and Figure 9 show a similar analysis for cells treated with SAHA (Figure 8) and TUBA (Figure 9), as well as literature-determined relationships between HDACs that are most affected by these inhibitors and the related subset of metabolites. Literature searches were performed using Pathway Studio software (Elsevier Inc., Ariadne Genomics, MD, USA).

Treatment with the pan-HDAC inhibitor, SAHA, does not cause significant metabolic profile changes in U373 cells, particularly in the intracellular metabolites, where only the concentration of isocitrate appears to be significantly affected across biological replicates. There are some changes in the extracellular metabolic profiles of U373 cells, however. This suggests that, based on the subset of metabolites observable by NMR, metabolism in U373 cells remains relatively unaffected by SAHA treatment. However, this is accomplished through alterations in metabolite exchange with the extracellular medium (Figure 4B), where concentrations of several metabolites show significant change in SAHA-treated U373 cells. HDAC inhibition by SAHA causes major metabolic profile changes in LN229 cells, both internally and externally (Figure 4A,B). Intracellular profiles show elevated lactate production, as well as higher levels of glucose and TCA cycle intermediates (Figure 8A). Previous analysis of the HDAC inhibition effects on metabolism has shown reduced glycolysis leading to higher levels of glucose, as well as increased amino acid catabolism and mitochondrial activity [26]. Overconcentration of TCA cycle intermediates, succinate, isocitrate and aconitate, can be due to glutamate and glutamine utilization as TCA carbon sources. Proline has been indicated as a significant factor linking metabolism and epigenetics [61], and therefore, it is interesting to observe an increased proline concentration in the extracellular media of both U373 and LN229 cells treated with SAHA (Figure 8), as well as TUBA (Figure 9). It is also important to point out that reduced intracellular glycine concentration is observed in all cells treated with HDAC inhibitors. Glycine synthesis has been identified as essential for tumour growth [62], thus pointing to another important effect of HDAC inhibition. Previous experiments in breast cancer cell lines have shown that inhibition of HDACs induces choline uptake and leads to elevated phosphocholine levels, due to increased SLC44A1 expression. This transporter is already significantly upregulated in LN229 cells [34] and, with the known downregulation of EGFR, provides an opportunity for more transport through the cell membrane for choline. With choline transport pathways being already overactive, SAHA treatment does not cause any significant change in glycerophosphocholine or choline in LN229 cells; instead, it leads to a reduced phosphocholine concentration, possibly due to changes in fatty acid metabolism. SAHA treatment leads to glutamine and glutamate depletion in cells without major changes in the extracellular medium, indicating an increased use, but not transport, of glutamine.

TUBA exposure also induces a reduction in phosphocholine levels in LN229 cells. At the same time, the increase in glycerophosphocholine in these same cell lines is significant. Similarly, as with SAHA treatment, glutamine and asparagine levels are reduced with TUBA treatment, possibly suggesting their use for fueling the TCA cycle. HDAC6, through its Hsp90 activation, regulates mitochondrial enzymes [28] and the inhibition of HDAC6 causes a reduction in the activity of respiratory complex II and citrate synthase. Our data (Figure 9D) suggest that, following HDAC6 inhibition, LN229 cells utilize glutamine as TCA cycle fuel, leading to increased isocitrate and aconitate concentrations. Citrate levels are reduced with citrate synthase deactivation. At the same time, pyruvate consumption is increased for lactate production with, most likely, a corresponding accumulation of acetyl-CoA and oxaloacetate (following citrate synthase deactivation).

Figure 10 represents the most significantly changed metabolites for treatments with i10, i12 and i8. According to PCA analysis (Figure 4), these treatments cause major changes in LN229 cells. Therefore, in Figure 10, we show only the major metabolic changes in LN229 cells treated with i10, i12 and i8. Figure 10 shows major metabolic differences in hydrophilic intracellular (A) and extracellular metabolites (B).

Significant changes in choline metabolism are observed following treatments with the novel HDAC inhibitors i12, i10 and i8. All treatments lead to a reduction in phosphocholine levels and an increase in glycerophosphocholine levels similarly to TUBA. In addition, there is a clear increase in glycerol-3-phosphate in all treatments, likely related to fatty acid biosynthesis, as discussed previously. Treatments with i10 in particular, but also with i12 and i8 to a lesser extent, lead to a large number of metabolic changes, probably due to their larger effect on HDAC6 than that provided by SAHA, coupled with more potent inhibition of other HDACs than TUBA. These larger metabolic effects can support their more significant influence on cell survival with the concentrations used (Figure 3) when compared with the three other tested compounds.

Changes in intracellular metabolic profiles can also be related to altered pH levels, and this would affect the activity of TMZ [63]. Inhibition of HDACs can lead to the reduction in pH [64], which would lead to reduced TMZ degradation into the active compound, possibly also resulting in increased cell survival with combined HDACi and TMZ treatment. However, treatments with compounds i8, i10 and i12 induce a significant increase in leucine concentration. Leucine has been indicated in previous work as a factor involved in increasing cytoplasmic pH level [65], possibly increasing the activity of TMZ. The exact connection between the effects of compounds i10 and i12 and TMZ will be a focus for future efforts.

## 3. Experimental Section

### 3.1. Cell Culture and Treatment Procedures

Human glioma cells LN229 and U373 were maintained in DMEM supplemented with 10% FBS (foetal bovine serum) and antibiotics (Invitrogen). Both cell lines were a kind gift of Adrian Merlo (Laboratory of Molecular Neuro-oncology, University of Basel, Basel, Switzerland) and have been described elsewhere [66]. All experiments were performed as three independent replicates (3 biological replicates). The 1 × 10^6^ cells were seeded in triplicates in 10-cm culture dishes, treated with DMSO (control), compounds, as well as compounds + TMZ and incubated for 72 hrs at 37 °C and 5% CO2. The DMSO concentration in cell media was 10 μM in all cases (to 200 μL total volume; in the cell cultures, we have added 0.9 μL of DMSO only or DMSO solution of treatment compounds corresponding to final concentration of to 10 μM or ~0.45% DMSO in cultures).

### 3.2. Crystal Violet Assay Procedure

A crystal violet cytotoxic assay was performed to assess the cytotoxic properties of the different compounds towards GBM cells. In addition, IC_50_ values for the inhibitors were measured in U373 cells with the same assay. Briefly, 10,000 cells were seeded in triplicates in 96-well plates in 200 μL of cell medium containing the inhibitors. Inhibitors were replaced by DMSO in control wells. Cells were incubated for 72 hrs at 37 °C and 5% CO2. Following incubation, the medium was aspirated and cells were rinsed with 200 μL of PBS. Cells were then fixed with 100 μL of cold methanol for 10 min. Cells were subsequently washed with 200 μL of PBS and stained with crystal violet for 20 min. Crystal violet was aspirated and excess dye washed with 200 μL of water. Stained cells were re-suspended in 100 μL of 1% SDS, and the absorbance of the solution was recorded at 595 nm on a Varioscan (Scanlab Inc., Munich, Germany). Viable cells were calculated as a percentage of absorbance with respect to DMSO control cells. Presented are the mean values with SEM.

### 3.3. IC_50_ Calculations Methodology

IC_50_ values for the inhibitors were measured in U373 cells using the crystal violet assay, as described above. To assess the IC_50_ values, molecules were also tested at different concentrations. All data are expressed as the mean of three experiments. IC_50_ values were calculated from a sigmoidal concentration-response curve-fitting model with a variable slope using GraphPad Prism 5 software (GraphPad Software, San Diego, CA, USA).

### 3.4. Intracellular Hydrophilic Phase Metabolites Isolation Procedure

Hydrophilic metabolite isolation was performed as described previously [34,47,48,56] in triplicates. Cells were harvested by scraping and rinsed with 5 mL of PBS. The mixture was centrifuged at 4000 RPM for 1 min. The supernatant was discarded and the cell pellet was rinsed with 4 mL of PBS. Following another centrifugation at 4,000 RPM for 1 min, the cell pellets were kept on ice for 5 min before being re-suspended in 1 mL of ice-cold 50% acetonitrile. Cell suspensions were kept on ice for 10 min before centrifugation at 16,000 RPM for 10 min at 4 °C. The aqueous acetonitrile extract supernatants were dried under a stream of N2.

### 3.5. Extracellular Metabolites Isolation Procedure

To isolate extracellular metabolites, 500 μL of extracellular medium were pipetted from compound-treated U373 and LN229 cells. The samples were subsequently centrifuged at 200 RCF for 5 min. The supernatant was used as the extracellular fraction.

### 3.6. NMR Experimentation and Preprocessing

The residue obtained after drying was dissolved in 0.6 mL of deuterium oxide (99.96 atom% 2H, Aldrich, Milwaukee, WL, USA) and pipetted into a 5-mm NMR tube for NMR analysis. All ^1^H NMR measurements were performed on a Bruker Avance III 400 MHz spectrometer at 298 K.

1D spectra were obtained using a gradient water presaturation method (pulse sequence zgesgp) with 512 scans [34], NMR spectra being processed using: Mnova 6.0.4 with exponential apodization (exp 1); global phase correction; Bernstein-polynomial baseline correction; Savitzky–Golay line smoothing; and normalization using the total spectral area, as provided in Mnova. Spectral regions from 0–9 ppm were included in the normalization and analysis; however, regions from 2.55–2.75 ppm and 1.816–1.848 ppm were cut, due to possible solvent contamination. Data pre-processing, including data organization, removal of undesired areas and binning, as well as data presentation was performed with Matlab [67]. Minor adjustments in peak positions (alignment) between different samples were performed using in-house alignment software (GASP), as well as Icoshift [68]. The qualitative analyses of the major variances in the spectra were performed by using principal component analysis (PCA), as well as fuzzy k-means cluster analysis using the MATLAB platform. Feature selection was performed with the Significance Analysis for Microarrays (SAM) method [57].

### 3.7. Metabolite Quantification

Peak assignment was performed using several methods developed in our group and elsewhere and was based on metabolic NMR databases [34,69,70]. Spectra for around 40 metabolites used in quantification were obtained from the Human Metabolomics Database [69] or the Biological Magnetic Resonance Databank [70] and were further analyzed visually and compared to the obtained spectra. An automated method for quantification based on multivariable linear regression of spectra with appropriately aligned metabolite data from databases was previously described in detail [34] and was used in this study. The assumption behind this approach is that the spectrum of a mixture is the same as the combination (sum) of spectra of individual components measured under the same conditions. Relative metabolite concentrations were estimated using nonlinear curve-fitting with the multivariate least-squares approach. The linear regression result was used as the starting point, and the model was constrained to concentrations: c ≥ 0. The NMR spectra of the mixtures (samples) are modeled as a sum of spectra for components (metabolites) in the mixture. Metabolite concentrations have been normalized across samples (assuming equal total metabolite concentration). For visualization, all samples were scaled across metabolites. All metabolite concentrations for all experiments are available from the authors.

## 4. Conclusions

In this work, we have investigated the effects of HDAC and SIRT inhibitors on the metabolism of two distinct GBM cell lines. Our results show interesting effects of inhibitors on mitochondrial metabolism, glycolysis and fatty acid synthesis. HDAC and SIRT inhibitors affected two cell cultures differently in terms of cell survival and metabolic shift. The addition of HDAC or SIRT inhibitors to TMZ treatment increased cell death in TMZ resistant lines (cell line LN229). However combining SAHA, TUBA or nicotinamide with TMZ treatment of the LN229 line increased the survival of these TMZ susceptible cells. Therefore, the addition of HDAC and SIRT inhibitors to the current GBM treatment regime could be highly beneficial in the therapy of some selected GBM subtypes, but irrelevant or even detrimental in other subtypes, making subtyping a necessary part of tumour treatment optimization. Metabolic differences between the two subtypes studied here can be used for the future development of biomarkers for treatment planning. Remarkably, novel compounds i10 and i12 increased the effectiveness of TMZ in both cell lines, therefore justifying further investigation of their possible application. In the future, we will test the effects of HDAC and SIRT inhibitors on a larger number of tumour cell models in an attempt to validate the hypotheses developed here, as well as to better identify specific pathways underlying the observed metabolic changes. From this approach, it will be possible to obtain valuable metabolic markers for HDACi or SIRTi treatment planning and follow-up.
